# Complete Genome Sequences of *Escherichia coli* O157:H7 Strains SRCC 1675 and 28RC, Which Vary in Acid Resistance

**DOI:** 10.1128/genomeA.00743-16

**Published:** 2016-07-28

**Authors:** Gian Marco Baranzoni, Pina M. Fratamico, Erin R. Reichenberger, Gwang-Hee Kim, Frederick Breidt, Kathryn Kay, Deog-Hwan Oh

**Affiliations:** a U.S. Department of Agriculture, Agricultural Research Service, Eastern Regional Research Center, Wyndmoor, Pennsylvania, USA; b USDA-Agriculture Research Service, SAA Food Science Research Unit, Raleigh, North Carolina, USA; c Department of Microbiology, North Carolina State University, Raleigh, North Carolina, USA; d Department of Food Science and Biotechnology, College of Bioscience and Biotechnology, Kangwon National University, Chuncheon, Republic of Korea

## Abstract

The level of acid resistance among *Escherichia coli* O157:H7 strains varies, and strains with higher resistance to acid may have a lower infectious dose. The complete genome sequences belonging to two strains of *Escherichia coli* O157:H7 with different levels of acid resistance are presented here.

## GENOME ANNOUNCEMENT

*Escherichia coli* O157:H7 strains are foodborne pathogens characterized by a low infectious dose and the ability to withstand low-pH environments. This allows *E. coli* O157:H7 to overcome the gastric barrier and survive in acidic foods, raising food safety concerns (1–3). *E. coli* acid resistance has been characterized primarily in reference strains and has been shown to be mediated by numerous systems, with an intricate regulation network (4, 5). Recently, it was shown that the ability to survive in acidic environments varies among *E. coli* strains belonging to the same serogroup isolated from different sources (6, 7).

In this work, we present the complete genome sequences of two *E. coli* O157:H7 strains with significantly different levels of acid resistance. The strains SRCC 1675 (isolate B0201) and 28RC1 (isolate B0241) were sampled from apple cider and a bovine carcass, respectively (6). Under the conditions tested, strain SRCC 1655 was less acid resistant than 28RC1 (6). The sequences were obtained by combining Ion Torrent (Thermo Fisher, Carlsbad, CA, USA) and PacBio (Pacific Biosciences, Menlo Park, CA, USA) technologies.

Ion Torrent libraries were prepared from genomic DNA extracted with the PureLink genomic DNA minikit (Thermo Fisher), using the Ion Xpress Plus fragment library kit (Thermo Fisher). Genomic libraries were enriched using the Ion PGM template OT2 400 kit (Thermo Fisher) on the Ion OneTouch 2 system (Thermo Fisher) and were sequenced using the Ion Torrent PGM on a 318 Chip. SMRTbell libraries (Pacific Biosciences) for single-strand sequencing were constructed from genomic DNA using Qiagen Genomic-tip 500/G columns (Qiagen, Valencia, CA, USA) and sequenced with P4-C2 chemistry on a PacBio RSII platform (Pacific Biosciences). One single-molecule real-time (SMRT) cell was used for each *E. coli* strain, with one 120-min movie. All kits were used according to the manufacturers’ protocols.

Two contigs were obtained for strains SRCC 1675 and 28RC1 from *de novo* genome assembly of PacBio reads using SMRT Analysis version 2.3.0 (average coverage, 102.0× and 107.5×, respectively). Variants of these assemblies were controlled by mapping Ion Torrent reads using TMAP (https://github.com/iontorrent/TMAP). Ion Torrent reads were *de novo* assembled using CLC Genomics Workbench version 7.5 (Qiagen), with default parameters. The resulting assemblies, along with the reference strain *E. coli* O157:H7 EDL933 (accession numbers CP008957.1 and CP008958.1), were aligned using MAUVE version 2.4.0 (http://darlinglab.org/mauve/mauve.html). In the 28RC1_plasmid, a gap of 11,469 bases was filled with Ion Torrent data. Another gap of 468 bases was filled in SRCC 1675 by Sanger sequencing. Annotation was performed using the NCBI Prokaryotic Genome Annotation Pipeline (https://www.ncbi.nlm.nih.gov/genome/annotation_prok/).

Based on the sequence data, the *E. coli* O157:H7 strain SRCC 1675 genome is composed of a chromosome and a plasmid spanning 5,506,801 bp and 95,170 bp, respectively; while the *E. coli* O157:H7 strain 28RC1 genome is composed of a chromosome and a plasmid spanning 5,561,698 bp and 81,401 bp, respectively. Both genomes have an overall G+C content of 50.5%.

### Nucleotide sequence accession numbers.

Nucleotide sequences were submitted to GenBank under the accession numbers CP015020 (28RC1_chromosome), CP015021 (28RC1_plasmid), CP015022 (SRCC 1675_plasmid), and CP015023 (SRCC 1675_chromosome). Raw data are publicly available in the Sequence Read Archive.

## References

[1] Price SB, Wright JC, Degraves FJ, Castanie-Cornet MP, Foster JW 2004 Acid resistance systems required for survival of *Escherichia coli* O157:H7 in the bovine gastrointestinal tract and in apple cider are different. Appl Environ Microbiol 70:4792–4799. doi:10.1128/AEM.70.8.4792-4799.2004. 15294816 PMC492388

[2] Bergholz TM, Whittam TS 2007 Variation in acid resistance among enterohaemorrhagic *Escherichia coli* in a simulated gastric environment. J Appl Microbiol 102:352–362. doi:10.1111/j.1365-2672.2006.03099.x. 17241340

[3] Kanjee U, Houry WA 2013 Mechanisms of acid resistance in *Escherichia coli*. Annu Rev Microbiol 67:65–81. doi:10.1146/annurev-micro-092412-155708. 23701194

[4] Foster JW 2004 *Escherichia coli* acid resistance: tales of an amateur acidophile. Nat Rev Microbiol 2:898–907. doi:10.1038/nrmicro1021. 15494746

[5] Zhao B, Houry WA 2010 Acid stress response in enteropathogenic *Gammaproteobacteria*: an aptitude for survival. Biochem Cell Biol 88:301–314. doi:10.1139/o09-182. 20453931

[6] Oh D-H, Pan Y, Berry E, Cooley M, Mandrell R, Breidt F 2009 *Escherichia coli* O157:H7 strains isolated from environmental sources differ significantly in acetic acid resistance compared with human outbreak strains. J Food Prot 72:503–509. 19343937 10.4315/0362-028x-72.3.503

[7] Kim G-H, Breidt F, Fratamico P, Oh D-H 2015 Acid resistance and molecular characterization of *Escherichia coli* O157:H7 and different non-O157 Shiga toxin-producing *E. coli* serogroups. J Food Sci 80:M2257–M2264. doi:10.1111/1750-3841.12996. 26375176

